# Alzheimer’s Caregiver Psychoeducation‐Based Psychological Support Telerehabilitation

**DOI:** 10.1002/alz.095661

**Published:** 2025-01-09

**Authors:** İdil Arasan Doğan, Feride Gökben Hızlı Sayar

**Affiliations:** ^1^ Uskudar University, Istanbul, Uskudar Turkey; ^2^ Uskudar University, İstanbul, Uskudar Turkey

## Abstract

**Background:**

Alzheimer’s Caregiver Psychoeducation‐Based Psychological Support Telerehabilitation; is an online program where the caregiver is educated about the disease and processes and supported therapeutically. This program also aims to promote the psychological well‐being of the caregiver and ensure a positive prognosis.

**Method:**

The study was conducted with 20 caregivers (relatives of patients) who applied to various branches of the Alzheimer Association of Turkey and received services, in a group setting using the Zoom Platform online. The control group consisted of 20 people who attended the association’s routine information meetings. Data collection tools were determined as the Sociodemographic Form, Brief Symptom Scale, Psychological Resilience Scale, and Zarit Caregiver Burden Scale. The study, based on psychoeducation, utilized Cognitive Behavioral Therapy Techniques and Positive Psychology Positive Interventions as therapeutic methods. The program was structured to last 6 weeks, with 12 sessions, twice a week for 90 minutes each. The telerehabilitation applied in a group setting was also supported with individual sessions. One support session per month was organized for 6 months following the completion of the program.

**Result:**

The age distribution of the caregivers was predominantly in the 45‐54 range, with a majority being female. In the experimental group, the scores for psychological symptoms such as Depression, Anxiety, Negative Self, Somatization, and Hostility in the final test were significantly lower compared to the pre‐test. No significant difference was observed in this context in the control group. Zarit Caregiver Burden scores in the experimental group also significantly decreased compared to the pre‐test. Regarding Psychological Resilience scores in the experimental group, the final test results showed an increase in psychological resilience levels.

**Conclusion:**

As a result of the implementation of the telerehabilitation program, caregivers' knowledge and awareness of Alzheimer’s Disease and the care process increased, their psychological resilience was strengthened, and their social support networks expanded. Additionally, the caregivers' burden of care decreased, and symptoms of depression and anxiety were alleviated. These findings emphasize the importance of accessible, applicable, and sustainable telerehabilitation programs.

